# Gustavson syndrome is caused by an in-frame deletion in *RBMX* associated with potentially disturbed SH3 domain interactions

**DOI:** 10.1038/s41431-023-01392-y

**Published:** 2023-06-05

**Authors:** Josefin Johansson, Sarah Lidéus, Carina Frykholm, Cecilia Gunnarsson, Filip Mihalic, Sanna Gudmundsson, Sara Ekvall, Anna-Maja Molin, Mai Pham, Mauno Vihinen, Kristina Lagerstedt-Robinson, Ann Nordgren, Per Jemth, Adam Ameur, Göran Annerén, Maria Wilbe, Marie-Louise Bondeson

**Affiliations:** 1 Department of Immunology, Genetics and Pathology, Biomedical Centre, Uppsala University, Uppsala, Sweden; 2 Department of Clinical Genetics, and Department of Biomedical and Clinical Sciences, Linköping University, Linköping, Sweden; 3 Centre for Rare Diseases in South East Region of Sweden, Linköping University, Linköping, Sweden; 4 Department of Medical Biochemistry and Microbiology, Uppsala University, Box 582, Husargatan 3, 751 23 Uppsala, Sweden; 5 Department of Experimental Medical Science, BMC B13, Lund University, SE-22 184 Lund, Sweden; 6 Clinical Genetics, Karolinska University Hospital, Solna, Sweden; 7 Department of Molecular Medicine and Surgery, Karolinska Institutet, Stockholm, Sweden; 8 Department of Clinical Genetics and Genomics, Sahlgrenska University Hospital, Gothenburg, Sweden; 9 Institute of Biomedicine, Department of Laboratory Medicine, University of Gothenburg, Gothenburg, Sweden; 10 Present Address: Program in Medical and Population Genetics, Broad Institute of MIT and Harvard, Cambridge, MA USA; 11 Present Address: Division of Genetics and Genomics, Boston Children’s Hospital, Boston, MA USA

**Keywords:** Medical genetics, DNA sequencing

## Abstract

RNA binding motif protein X‐linked (*RBMX*) encodes the heterogeneous nuclear ribonucleoprotein G (hnRNP G) that regulates splicing, sister chromatid cohesion and genome stability. *RBMX* knock down experiments in various model organisms highlight the gene’s importance for brain development. Deletion of the RGG/RG motif in hnRNP G has previously been associated with Shashi syndrome, however involvement of other hnRNP G domains in intellectual disability remain unknown. In the current study, we present the underlying genetic and molecular cause of Gustavson syndrome. Gustavson syndrome was first reported in 1993 in a large Swedish five-generation family presented with profound X-linked intellectual disability and an early death. Extensive genomic analyses of the family revealed hemizygosity for a novel in-frame deletion in *RBMX* in affected individuals (NM_002139.4; c.484_486del, p.(Pro162del)). Carrier females were asymptomatic and presented with skewed X-chromosome inactivation, indicating silencing of the pathogenic allele. Affected individuals presented minor phenotypic overlap with Shashi syndrome, indicating a different disease-causing mechanism. Investigation of the variant effect in a neuronal cell line (SH-SY5Y) revealed differentially expressed genes enriched for transcription factors involved in RNA polymerase II transcription. Prediction tools and a fluorescence polarization assay imply a novel SH3-binding motif of hnRNP G, and potentially a reduced affinity to SH3 domains caused by the deletion. In conclusion, we present a novel in-frame deletion in *RBMX* segregating with Gustavson syndrome, leading to disturbed RNA polymerase II transcription, and potentially reduced SH3 binding. The results indicate that disruption of different protein domains affects the severity of *RBMX*-associated intellectual disabilities.

## Introduction

RNA binding proteins (RBPs) are ubiquitously expressed regulators of RNA processing, especially important for post-mitotic neurons to control temporal individual differences in axonal growth, plasticity, and function [1]. Heterogeneous nuclear ribonucleoproteins (hnRNPs) belong to the RBP family of structurally related proteins, recently highlighted in Gillentine et al. 2021, as not fully investigated candidate genes for neurodevelopmental disorders (NDDs). NDDs associated with hnRNPs have many shared phenotypes including severe structural brain abnormalities, intellectual disability, seizures, speech delay and hypotonia, and are suggested to share a common molecular pathogenesis [2]. RNA binding motif protein X‐linked (*RBMX*) encodes hnRNP G important for splicing regulation [3], sister chromatid cohesion regulation [4] and genome stability [5] specifically in neurons [6]. hnRNP G is part of the supraspliceosome, previously reported to be important for alternative splice site selection by protein binding (C-terminal) and RNA binding (RNA recognition motif;RRM) [3]. Overexpression of *RBMX* leads to exon skipping or inclusion where hnRNP G-dependent exons are significantly enriched in CCA/CCC motifs, suggesting a function in exon skipping/exon inclusion regulation [3]. *RBMX* morpholino knock down in zebrafish [7], African frog [8] and neuronal in-vitro studies [9, 10] highlight the gene’s importance for brain development and function. Moreover, a 23 bp frameshift deletion disrupting the *RBMX* RGG/RG motif in the RNA binding domain is associated with a mild to moderate X-linked intellectual disability (XLID) called Shashi syndrome (OMIM #300238) [10, 11]. However, the involvement of other hnRNP G domains in intellectual disability remains unknown.

Gustavson syndrome was first described in 1993 in six males and one female of a Swedish five-generation family (OMIM #309555) [12, 13]. The syndrome was characterized by profound intellectual disability, microcephaly, severe structural brain abnormalities, epileptic seizures, severe vision defect, hearing loss, congenital heart defects, psychomotor deficits, and an early death before 4 years of age due to pulmonary infections. Linkage analysis of 21 affected and unaffected family members indicated an association to the Xq26 region, including *RBMX* and 399 other genes, and an X-linked recessive inheritance pattern [13]. However, the genetic variant/s causing Gustavson syndrome was not established at that time.

In this study, we investigated the underlying genetic and molecular cause of Gustavson syndrome. Three additional affected family members have been identified since 1993 and presented striking phenotypic overlap with previously described patients. Genomic analyses of 36 family members revealed hemizygosity for an in-frame deletion in *RBMX* (NM_002139.4; c.484_486del, p.(Pro162del)) that segregated with the disease in the large family pedigree. Asymptomatic heterozygous females presented with a skewed X-chromosome inactivation (XCI) pattern, indicating silencing of the pathogenic allele. Transcriptomics and differential gene expression analysis of neuronal cells harboring the variant revealed that the top 100 differentially expressed genes (DEGs) were significantly enriched for transcription factors and genes involved in RNA polymerase II transcription, highlighting hnRNP Gs important role during transcription. Variant predictions and fluorescence polarization assay show that the variant is located in a highly conserved region which is likely to be a novel polyproline II helix/Src homology-3 (SH3) binding domain of hnRNP G. The fluorescence polarization assay indicates that the variant potentially lowers the binding affinity to proteins with SH3 domains.

## Materials and methods

### Patient samples

**Fig. 1:**
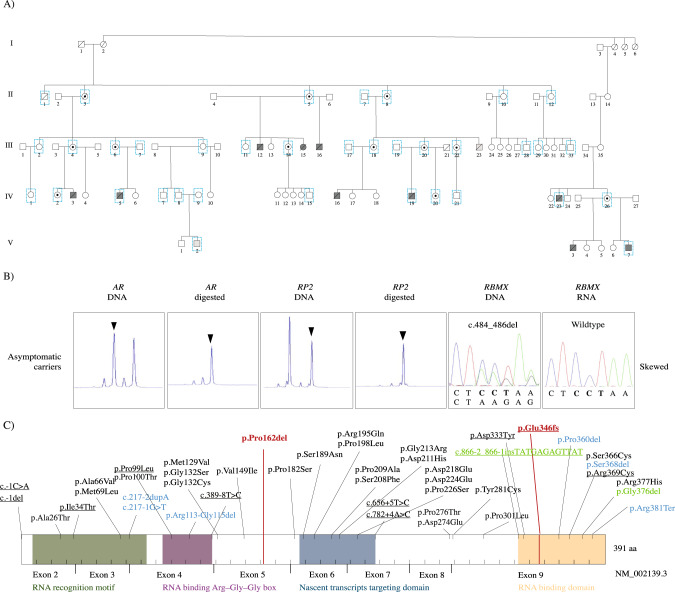
Genetic and molecular characterization of Gustavson syndrome. **A** Extended family pedigree of the family affected with Gustavson syndrome (91 individuals). Sanger sequencing of the *RBMX* variant in 36 individuals (blue squares) revealed that all investigated affected individuals were hemizygous (dark gray) and had inherited the allele from their carrier mothers. Patients with trisomy 18 is highlighted in light gray. **B** X-chromosome inactivation (XCI) analysis and variant detection on DNA and RNA confirms that asymptomatic carrier females (*n* = 11) are protected from disease by silencing the pathogenic allele (black arrow). XCI result from all individuals are presented in Supplementary Fig. 1. **C** Schematic of hnRNP G with known domains. Top: *RBMX* variants listed in the gnomAD and ClinVar databases. ClinVar variants are underlined, green: homozygous/hemizygous variants (all reported stop gained, start lost, splice acceptor, in-frame insertions/deletions or frameshift insertions/deletions), blue: heterozygous variants (all reported stop gained, start lost, splice acceptor, in-frame deletions or frameshift deletions), black: homozygous/hemizygous missense variants. Red lines: variants associated with Gustavson syndrome (p.(Pro162del)) and Shashi syndrome (p.Glu346fs). GnomaD variants obtained 22-07-28, including variants in the canonical transcript and excluding variants flagged as of poor quality or in low complexity regions. Bottom: Domains of hnRNP G are RNA recognition motif, RNA binding Arg-Gly-Gly-box, Nascent transcripts targeting domain, RNA binding domain (52, 54).

The investigated Swedish five-generation family consist of 91 individuals (Fig. 1A). Ten individuals (III:12, III:15, III:16, IV:3, IV:5, IV:16, IV:19, IV:23, V:3, V:7) were affected with Gustavson syndrome. Thirty-six individuals of the extended pedigree were included in the genetic analysis. DNA and RNA were extracted from blood by standard procedures (available upon request).

### Genetic characterization of Gustavson syndrome

#### Genome sequencing by Illumina

Genome sequencing and analysis was performed on two family trios (III:19, III:20, IV:19 or IV:26, IV:27 and V:7) by two independent labs. The first trio (III:19, III:20, IV:19) were sequenced from 1 μg DNA using the TruSeq PCRfree DNA sample preparation kit (350 bp, cat.20015962/3, Illumina) and library prepped according to the manufacturers’ instructions (guide#1000000039279). Sequencing was performed on a NovaSeq S4 flowcell, with v1 sequencing chemistry, paired-ends and 150 bp read length. Alignment to reference genome (hg19) was performed using BWA-MEM and variant calling was performed using GATK 3.3.0. Manta Structural Variant Caller 1.0.3 was used to identify structural variants, and small insertions and deletions [14]. Variants were analyzed using MOON software (www.diploid.com/moon) with the filtering criteria: depth ≥8, genotype quality ≥40 and allele frequency ≤1.0% in gnomAD and Diploid database (www.diploid.com/moon). The other trio (IV:26, IV:27 and V:7) was sequenced and bioinformatically analyzed as previously reported [15]. All inheritance patterns were considered. All SNVs and SVs segregating with Gustavson syndrome in the extended pedigree were considered for further analysis.

#### Genome sequencing by 10X Genomics

Linked-read barcoded sequencing libraries were prepared from 1.25 ng DNA of a family trio (III:19, III:20, IV:19) using the Chromium Genome reagent kit v2 (cat.120257/58/61/62, 10X Genomics) according to the manufacturers’ protocol (#CG00043 Chromium Genome Reagent Kit v2 User Guide, 10X Genomics). Genome sequencing was performed on a NovaSeq 6000 S4 flowcell with v1 sequencing chemistry, paired-ends and a read length of 150 bp. The reads were mapped towards the reference genome hg38, and variants were called using LongRanger 2.2 (10X Genomics). Strict filtering was performed where large SVs (>30 kb) and deletions (50 bp-30 kb), shared in the mother and affected fetus were further investigated. Linked-read data were visualized using Loupe software 2.1.2 (10X Genomics). Variants were only considered if they segregated with Gustavson syndrome in the extended pedigree. Genome sequencing data from the family were used for segregation analysis. Variants were excluded if they were sequencing artefacts or present in our in-house database consisting of 1424 exomes from individuals with no signs of Gustavson syndrome [16].

#### In silico predictions of the p.(Pro162del) in RBMX

hnRNP G (P38159) 3D structure was predicted using AlphaFold2 [17]. The evolutionary conservation of the deleted bases of *RBMX* was investigated using phyloP and GERP scores. MutationTaster2 and PROVEAN were used to investigate the variant effect on protein function, SpliceAI and Human Splicing Finder were used to investigate the variant effect on alternative splicing. The genome databases gnomAD and SweGen [18] were used to investigate the variant landscape in *RBMX* present in the general population. Polyproline II secondary structures of hnRNP G (P38159) were predicted using the PPIIPRED software (http://bioware.ucd.ie/PPIIPRED) [19], and disordered regions were predicted using Database of Disordered Protein Predictions (D2P2) [20], and AlphaFold2 [17].

#### Segregation analysis

DNA was extracted from peripheral blood from 36 individuals in the extended pedigree (Fig. 1A and Supplementary Table 1). The region spanning the *RBMX* variant (NM_002139.4; c.484_486del, p.(Pro162del)) was amplified using 20–50 ng DNA, the RBMX_DNA primers and PCR protocol described in Supplementary material 1. Cycle sequencing was then performed using BigDye Terminator v3.1 Cycle Sequencing kit (Thermo Fisher Scientific, Carlsbad, CA, USA) according to manufacturer’s instructions with cycling parameters: 96°C 1 min, 30x (96°C 10 s, 50°C 5 s, 60°C 30 s) 4 °C hold. All samples were cleaned up using BigDye XTerminator Purification kit (Thermo Fisher Scientific, Bedford, MA, USA) and capillary electrophoresis was performed on 3130XL ABI Genetic Analyzer (Thermo Fisher Scientific, Foster City, CA, USA). The result was analyzed using CodonCode Aligner v9.0.1 (CodonCode Corporation, www.codoncode.com).

### Molecular characterization of Gustavson syndrome

#### X-chromosome inactivation analysis of healthy carrier females

XCI patterns were investigated in eleven asymptomatic carrier females aged 20–60 years (III:20, III:18, III:22, II:3, III:4, IV:2, II:5, III:6, III:14, IV:26, IV:20), six asymptomatic non-carriers (III:9, III:2, IV:2, IV:9, II:10, II:12) and seven controls. XCI analysis of the *AR* and *RP2* genes were performed using PCR and Fragment Length Analysis (FLA) as described before [21, 22]. Two hundred ng of genomic DNA were cut using HpaII FastDigest in a total volume of 20 µl following manufacturer’s instructions (Thermo Fisher Scientific, Baltics UAB, Vilnius, Lithuania). PCR was performed using 50 ng of DNA or 2 µl digested DNA as input, primers and PCR conditions as described in Supplementary material 1. FLA was performed on the 3130xl ABI Genetic Analyzer with ROX500 Size Standard (Thermo Fisher Scientific, Carlsbad, CA, USA) and the Amplified Fragment Length Polymorphism was determined using GeneMarker software v2.6.3 (SoftGenetics, State College, PA, USA). Polymorphic repeats in non-carrier females, parents and/or offspring from the family were used to indicate the pathogenic allele. The XCI result was confirmed with Sanger sequencing spanning the *RBMX* variant using cDNA synthesized from RNA blood (800 ng and 24 ng of RNA) of two carrier mothers (III:20, III:22) according to protocol (Maxima H Minus First Strand cDNA Synthesis Kit with dsDNase, Thermo Fisher Scientific, Waltham, MA, USA). PCR was performed as described in Supplementary material 1 using 50 ng cDNA and the primers RBMX_RNA.

#### RBMX splicing investigation

Due to the predicted variant effect on alternative splicing, a splicing analysis was performed on RNA extracted from peripheral blood of an affected individual (V:7). cDNA synthesis was performed on 2.2 µg RNA as described above. PCR was performed using 1 µl cDNA, primers spanning the whole gene (RBMX_Splicing_1 and RBMX_Splicing_2) and protocol described in Supplementary material 1. Sanger sequencing was performed using the Mix2seq kit (Eurofin Genomics, Ebersberg, Germany).

To confirm the result in other cell types, a mini-gene splicing assay was performed in HeLa cells and SH-SY5Y cells. The mini-gene construction, cell transfection, RNA extraction and cDNA synthesis were performed as described in Supplementary methods 1.

#### hnRNP G interaction study

To further investigate if the tri-proline region spanning the variant had the capacity to interact with SH3 domains, a fluorescence polarization assay with hnRNP G peptides and SH3 domains were performed. Wildtype and mutant (△P162) hnRNP G peptides of different lengths spanning the region of interest (P38159) were ordered from Genecast (Supplementary material 2) with or without Fluorescein isothiocyanate (FITC)-label (purity >95%, dissolved in 50 mM potassium phosphate pH 7.5). Two SH3 domains were expressed and purified: ASAP1-SH3 (Addgene: 91501), BIN1-SH3 (GenScript) according to standard protocol (Supplementary material 2).

To determine the affinity between the SH3 domains and the FITC-labeled hnRNP G peptides, saturation binding experiments were performed by preparing the 1:1 dilution series of increasing protein concentration (0.3–620 μM for ASAP1-SH3 and 0.425–870 μM for BIN1-SH3) and mixing it with a fixed concentration of FITC-labeled peptides (10 nM). Fluorescence polarization was measured with SpectraMax iD5 plate reader (Molecular Devices) at 25°C and excitation/emission wavelengths of 485/535 nm.

To determine the affinity between BIN-SH3 domain and the non-labeled hnRNP G peptides, displacement experiments were performed where the fixed concentration of FITC-labeled peptide and protein was mixed with an increasing concentration of displacer non-labeled peptide. The mP signal was investigated to determine the *IC*_*50*_ values, which were then converted to dissociation constants as previously described [23]. All results were analyzed with GraphPad Prism to determine the dissociation constant for labeled peptide and the *IC*_*50*_ values, and Microsoft Excel was used to calculate *K*_D_ from *IC*_*50*_. All fluorescence polarization experiments were performed in 50 mM potassium phosphate pH 7.5, containing 1 mM tris(2-carboxyethyl)phosphine (TCEP).

#### Transcriptomics on neuronal cells expressing the p.(Pro162del) in RBMX

Transient transfection was performed in SH-SY5Y cells using pcDNA3.1( + )-C-eGFP plasmid constructs containing *RBMX* cDNA sequence with and without the variant (c.484_486del, p.(Pro162del)) (Supplementary material 3). To control for technical bias, two independent cell and sequencing experiments were performed, one in duplicates and the other in triplicates. For each experiment, SH-SY5Y cells (passage 65 or 67) were cultured as described previously and 3.4 × 10^6^ cells were seeded to nine wells of 6-well plates. Transfection and RNA extraction was performed as previously described. Libraries were prepared using 260 ng of poly(A)-selected RNA with the TruSeq Stranded mRNA sample preparation kit according to the manufacturer’s protocol (Illumina Inc., San Diego, CA, USA). The quality of the libraries was evaluated using Agilent 2100 Bioanalyzer (Agilent, Santa Clara, CA, USA) using the RNA 6000 Nano reagent kit (Agilent, Hopkinton, MA, USA). RNA sequencing was performed on a SP flowcell using the NovaSeq 6000 system and v1.5 sequencing chemistry (Illumina Inc., San Diego, CA, USA) according to manufacturer’s instructions. Demultiplexing and conversion to FASTQ format was performed using bcl2fastq (v2.20.0.422). Trimmomatic (v0.39) was used for trimming adapter contamination [24], and quality was assessed using FastQC (v0.11.9) [25] and MultiQC (v1.12) [26]. STAR (v2.7.9a) was used for alignment to the reference genome (GRCh38.p13/hg38). Reads were counted to exons by using featureCounts (Rsubread v1.5.2) [27] followed by differential gene expression analysis with adjustment for batch effects using DESeq2 [28]. To identify enriched protein classes and pathways in the top 100 DEGs, PANTHER statistical overrepresentation test (v17.0) was used with correction for multiple testing using Bonferroni [29]. Data visualization was performed using Rstudio (v2021.09.1) and pheatmap with default settings (v1.0.12).

#### Protein expression and localization analysis

To investigate if the variant alters the location of hnRNP G a new transfection (SH-SY5Y cells, passage 69) was performed as previously described but using a µ-Slide 8 Well (ibidi, Gräfelfing, Germany). Images were taken 48 h post transfection using EVOS FL Auto 2 (Fisher Scientific, Willow Creek, Eugene, OR, USA) and protein expression was approximated using ImageJ (Rasband, W.S., ImageJ, U. S. National Institutes of Health, Bethesda, Maryland, USA, https://imagej.nih.gov/ij/, 1997–2018). A permutation test (*N* = 10,000) was performed to determine if the difference in mean values were significant (*p* < 0.05). Localization of hnRNP G was determined using a combination of GFP expression and DAPI staining. Imaging was performed using Zeiss LSM700 Confocal Microscope, at 40x magnification (Supplementary methods 3).

## Results

### Case reports of affected individuals

**Fig. 2:**
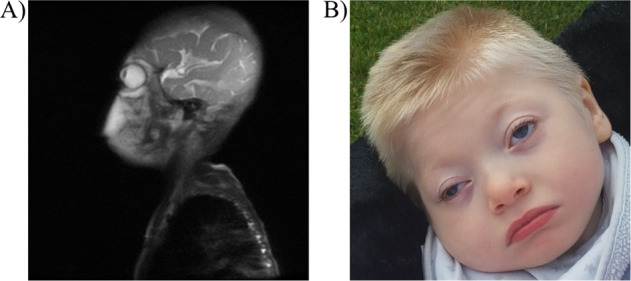
Clinical manifestations in patient V:7. **A** Magnetic resonance imaging showing lissencephaly with very thick cortex and dorsal dominance. As well as, some hypotrophy of vermis, while the frontal part of corpus callosum is well developed and the back part is thinner. **B** Patient V:7 showing dysmorphic features include bitemporal narrowing, wide mouth, puffy eyelids, broad mouth, high palate, two hemangiomas, microcephaly, short stature and overweight. For more detailed clinical description and phenotype comparison to other patients with Gustavson syndrome, see Supplementary data 1 and Supplementary Table 4B.

Six affected males (III:12, III:16, IV:3, IV:5, IV:16, IV:19) and one affected female (III:15) were first described in 1993 [19]. Since then, three additional affected males (IV:23, V:3, V:7) have been identified in this family, with striking phenotypic overlap to previously described affected individuals, including profound intellectual disability, seizures, microcephaly (Fig. 2; detailed case reports in Supplementary data 1).

### Genetic characterization of Gustavson syndrome

#### RBMX p.(Pro162del) segregates with Gustavson syndrome in the large family

Initially exome sequencing (Illumina) was performed on six family members (IV:5, III:6, IV:19, III:20, IV:26, V:7), but no candidate variants segregating with disease was identified. Then, genome sequencing was performed using Illumina and 10X Genomics sequencing. MOON software listed 60 variants following all inheritance patterns using Illumina genome sequencing. Loupe software detected 1262 deletions (50 bp-30 kb) and 41 large SVs (>30 kb) shared by the mother and child when using 10X Genomics sequencing. Both Illumina and Linked-read sequencing uncovered a variant of unknown significance (NM_002139.4; c.484_486del, p.(Pro162del)) in *RBMX* (RNA binding motif protein X-linked) located in the previously identified linked region Xq26. All variants except c.484_486del in *RBMX* were excluded as sequencing artefacts or because they did not segregate with disease (Supplementary Table 2). The result was confirmed by an independent second trio analysis in the same family. The variant is located in an evolutionary conserved locus (phyloP score 4.88, GERP 5.39). MutationTaster2 and PROVEAN suggest that the variant has a deleterious effect on protein function (PROVEAN score −8.666) and Human Splicing Finder predicted alternative splicing while SpliceAI did not. The c.484_486del variant is not present in public reference genomes (gnomAD, SweGen) and *RBMX* was reported to be depleted from pLoF variants (gnomAD pLI 0.83, o/e 0.14 CI 0.06–0.43) (Fig. 1C). Sanger sequencing of 36 samples from the family, spanning the identified *RBMX* variant confirmed that the variant segregated with Gustavson syndrome with an X-linked recessive inheritance pattern (Fig. 1A and Supplementary Table 1).

### Molecular characterization of Gustavson syndrome

#### Skewed X-chromosome inactivation in healthy carrier females

XCI analysis [21, 22] was performed using PCR and FLA spanning the *AR* and *RP2* genes in eleven carrier females, six non-carrier females and controls. Segregation of the polymorphic repeats in *AR* and *RP2* indicated skewed XCI (100:0), silencing of the pathogenic allele in carrier females (Fig. 1B, Supplementary Fig. 1B). FLA analysis of non-carrier females showed normal XCI patterns, consistent with previously published data of the general population (Supplementary Fig. 1C) [30]. RNA sequencing spanning the *RBMX* variant confirmed that XCI leads to silencing of the pathogenic allele in two carrier females by only expressing the wildtype allele (Fig. 1B).

#### RBMX splicing assay reveals no variant effect on alternative splicing

Due to a predicted effect of the c.484_486del variant on alternative splicing, we performed a *RBMX* splicing analysis on blood from an affected hemizygous individual and in SH-SY5Y cells and HeLa cells overexpressing the mutant *RBMX* mini-gene. Sanger sequencing of cDNA showed no splicing effect within *RBMX* when the variant was present in the investigated tissues (Supplementary Fig. 2).

#### The p.(Pro162del) in hnRNP G is located in a novel SH3-binding motif, and potentially reduces the binding affinity to SH3 domains

**Fig. 3:**
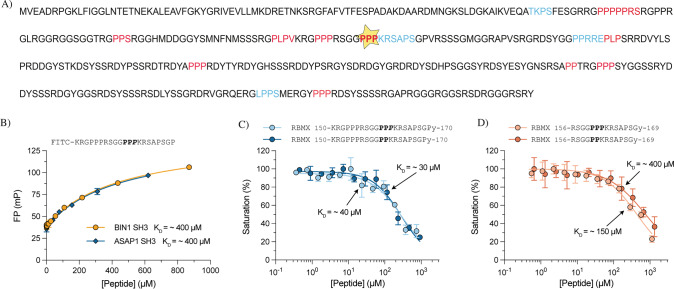
SH3-domain investigation in hnRNP G. **A** hnRNP G amino acid sequence (P38159) with the p.(Pro162del) variant highlighted with a star, predicted polyproline II helices highlighted in red (strong prediction) and blue (weaker prediction) using PPIIPRED prediction. **B** Saturation binding experiments between two SH3 domains and the labeled hnRNP G peptide (amino acids 150–170) revealed binding with moderate affinities to SH3 domains. **C** Displacement binding experiments between BIN1-SH3 domain and two longer hnRNP G peptides (wildtype and △P162 variant) showed no clear difference in SH3 binding affinity between the wildtype and the mutant peptide. **D** Displacement experiments between BIN1-SH3 and the shorter hnRNP G peptides (wildtype and △P162 variant), showing a reduced binding affinity to SH3 domains when the p.(Pro162del) variant was introduced.

The tri-proline region of hnRNP G (aa 160–162) was predicted to form a polyproline type-II helix structure (PPIIPRED aa 162;PPII score 0.66), located in a disordered region (aa 162;IUPred score 0.98, aa 160–162;AlphaFold2 pLDDT score 70, D2P2 consensus score) (Fig. 3A and Supplementary Fig. 3). Since polyproline II helices frequently bind to SH3 domains [31, 32], we further investigated if the tri-proline region had the capacity to interact with SH3 domains. Saturation binding experiments revealed binding to SH3 domains (BIN1 and ASAP1) with an affinity of 400 μM for a FITC-labeled peptide corresponding to residues 150–170 (Fig. 3B), and 40 μM for a non-labeled peptide (Fig. 3C), thus confirming SH3 domain binding capacity. To investigate if the putative SH3 recognition motif in the vicinity of the deletion is sufficient for the interaction [33], we measured the affinity for a shorter hnRNP G peptide (aa 156–169). The peptide showed weaker but comparable affinity for BIN1-SH3 domain (150 μM) (Fig. 3D), thus confirming the importance of the region. The adjacent N-terminal residues in hnRNP G (aa 150–170) contributed to the affinity.

The effect of the p.(Pro162del) on SH3-binding was tested both for the long and the short hnRNP G peptide described above. The short peptide (aa 156–169 △P162) displayed a threefold weaker affinity for BIN1-SH3 (K_D_ = 400 μM), while the long peptide (aa 150–170 △P162) showed no clear difference in binding affinity compared to the wildtype peptide (K_D_ = 30 μM) (Fig. 3).

#### Localization and expression of hnRNP G in neuronal cells

To investigate if the P162del variant affects the location and/or expression of hnRNP G, mutant and wildtype cDNA was transiently expressed in SH-SY5Y cells. Confocal fluorescent images showed no difference in location for hnRNP G between the cell-lines. Merged images of the GFP expression and DAPI show that hnRNP G is expressed in the nucleus (Supplementary Fig. 3A). Comparing the mean expression of the two populations resulted in a difference in intensity of 488.2 units. The permutation test resulted in a 95% confidence interval of [−2217.3, 2093.4], and indicate no significant difference in expression (*p* = 0.6641) (Supplementary Fig. 3B).

#### Transcriptomics of neuronal cells expressing the RBMX p.(Pro162del) variant

**Fig. 4:**
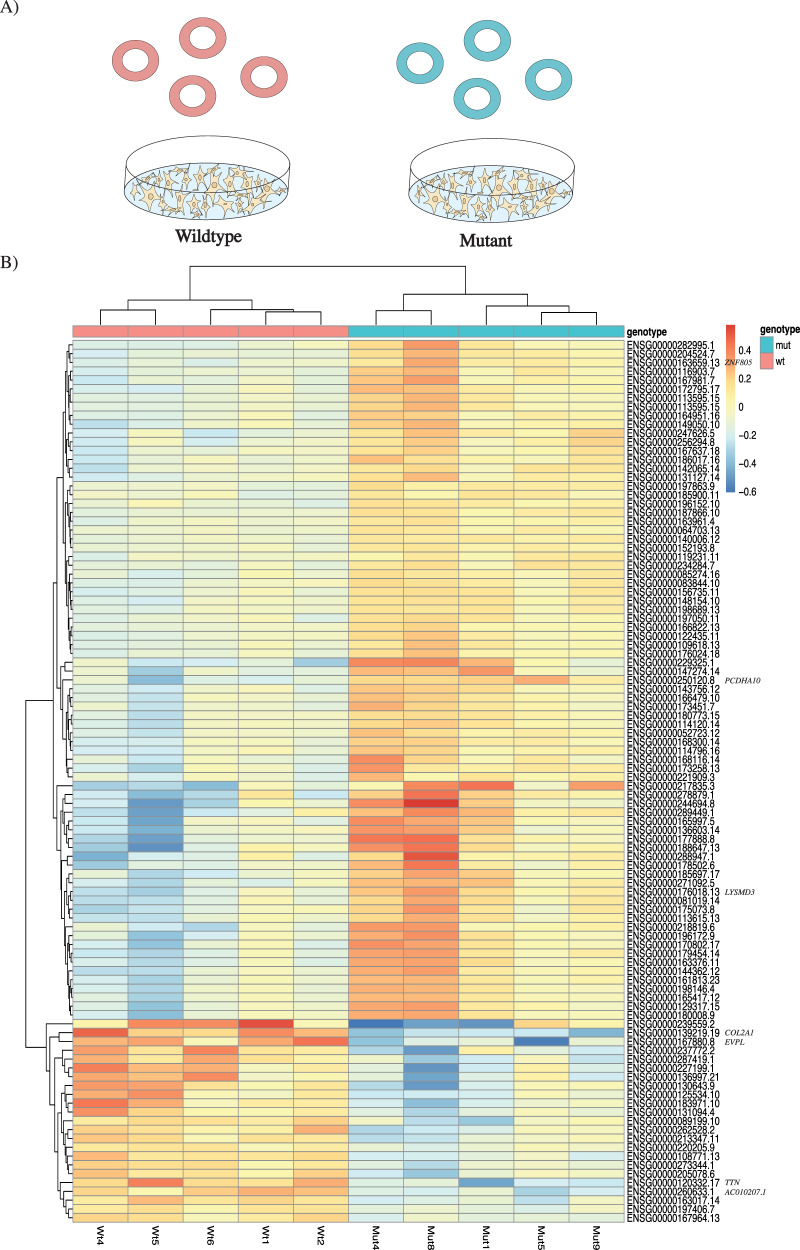
Transcriptome analysis of *RBMX* in neuronal cells. **A** Transcriptomics experimental setup with transient transfection of the wildtype vector (pink) or mutant vector (p.(Pro162del): turquoise) in SH-SY5Y cells. **B** The top 100 most differentially expressed genes between wildtype (pink) and mutant cells (turquoise). Each line represents the expression of one gene, where upregulated genes are red and downregulated genes are blue. The genes (rows) and samples (columns) of the top 100 most differentially expressed genes are hierarchical clustered according to expression patterns. Significantly upregulated genes (star) in the mutant cells (padj<0.05): *ZNF805*;ENSG00000204524.7, *PCDHA10*;ENSG00000250120.8, *LYSMD3*;ENSG00000176018.13. Significantly downregulated genes (star) in the mutant cells (padj<0.05): *COL2A1;*ENSG00000139219.19, *EVPL;*ENSG00000167880.8, *TTN*;ENSG00000120332.17*, AC010207.1*;ENSG00000260633.1.

Transcriptomics and differential gene expression analysis of neuronal cells expressing the variant revealed seven significant DEGs, where mutant cells presented a significant upregulation (padj<0.05) of *ZNF805*, *PCDHA10*, *LYSMD3* and downregulation of *COL2A1*, *EVPL*, *TTN*, *AC010207.1*. *EVPL* encodes for a protein with an SH3 domain (Supplementary Table 3). Hierarchical clustered heatmap visualization of the top 100 DEGs revealed a distinct difference in expression pattern when comparing the wildtype and mutant expression levels (Fig. 4). The top 100 DEGs were significantly enriched for transcription factors and genes in the RNA polymerase II transcription process (Supplementary Table 3B, C).

## Discussion

We have identified a novel in-frame deletion in *RBMX* (c.484_486del, p.(Pro162del)) leading to Gustavson syndrome (OMIM #309555) [12, 13] by disturbed RNA polymerase II transcription and potentially reduced SH3 binding. Linkage analysis previously mapped Gustavson syndrome to the Xq26 region, which includes *RBMX* and 399 other genes [13]. The *RBMX* variant was identified using genome sequencing, but missed during exome analyses due to poor alignment in a complex GC-rich region and presence of human retroposon-derived *RBMX*-like genes, emphasizing the usefulness of genome sequencing in *RBMX*-related disorders. XCI analysis of eleven carrier females showed skewed X-chromosome inactivation (100:0), indicating silencing of the pathogenic allele, and protection from disease. RNA analysis confirmed the XCI result by expression of only the wildtype allele. A crossover event in the Xq26 region, leading to exclusion of the *RBMX* variant, was observed in one non-carrier female (III:2) and her daughter (IV:1) (Supplementary Fig. 1) [13], strengthening the predicted pathogenic effect, since both were asymptomatic and the daughter (IV:1) had random X-inactivation pattern. Interestingly, a girl has been reported to suffer from Gustavson syndrome [12, 13]; however, due to lack of patient material further investigation of her is not possible, but could be caused by uniparental isodisomy, or an additional/alternative diagnosis such as Turner syndrome.

The variant p.(Pro162del) was predicted to have a deleterious effect on protein function and cause alternative splicing of *RBMX*. However, alternative splicing of *RBMX* caused by the variant was excluded as the mechanism of disease by no detected aberrant splicing events in RNA of an affected hemizygous individual, SH-SY5Y cells and HeLa cells overexpressing the mutant *RBMX* mini-gene. The variant is located in an evolutionary conserved locus (phyloP score 4.88, GERP 5.39) with an unknown function [34, 35]. The variant deletes a proline of a tri-proline structure, predicted to be a polyproline type-II helix [19]. Polyproline type-II helices are important for protein-protein interaction motifs especially for SH3 and Enabled/VASP Homology-1 domains [31, 32]. Polyproline type-II segments are often structurally flexible, with an important structure for protein folding [32], appearing within intrinsically disordered regions. The RBMX variant is located in a disordered region which has a flexible undefined structure depending on ligand binding and environment [17, 19, 20]. To our knowledge, the function of tri-prolines at amino acid residues 160–162 (hnRNP G; P38159) and protein interactions with this region, have not been published before.

To confirm the effect of the p.(Pro162del) variant on hnRNP G function, fluorescence polarization displacement experiments were performed. hnRNP G aa 156–169 was found to bind to BIN1-SH3 and ASAP1-SH3 domains with moderate affinities, thus confirming that this region of hnRNP G can bind to SH3 domains. However, residues 150–170 also contributed to the affinity. Published affinities of SH3 domains vary from low nanomolar to high micromolar [36, 37], and are affected by adjacent domains and motifs [33, 38]. Thus, the variant effect is context dependent and likely complex in vivo. The BIN1-SH3 and ASAP1-SH3 domains used in this experiment were chosen based on predicted specificities of recognition motifs [33]; however, they are likely not the natural cellular interaction partner/s of the hnRNP G SH3 binding motif. Further studies are needed to reveal the interaction partner/s in cells. In summary, our data suggest that residues 150–170 can interact with SH3 domains and the p.(Pro162del) variant may reduce the affinity.

Differential gene expression analysis on neuronal cells expressing the wildtype or mutant (c.484_486del;p.(Pro162del)) *RBMX* was performed. Transcriptomics analysis revealed seven significant DEGs with where mutant cells presented a significant downregulation of *EVPL*, *COL2A1*, *TTN*, *AC010207.1* and upregulation of *ZNF805*, *LYSMD3*, *PCDHA10*. The low number of significant DEGs is likely a consequence of few replicates, indicated by a clustered heatmap of the top 100 DEGs showing a distinct expression pattern among the genes in the two groups (Fig. 4). Top 100 DEGs revealed an overrepresentation of genes involved in RNA polymerase II transcription, and specifically genes encoding transcription factors. One of the significantly downregulated genes encodes a protein with a SH3 domain (*EVPL*), further supporting the possibility that the variant reduces SH3-binding affinity. We also show that these differences are not due to a difference in expression or localization of hnRNP G. *EVPL* encodes envoplakin, and *EVPL* -/- mice and zebrafish indicate a function in the skin barrier development [39, 40]. The SH3 domain in *EVPL* is highly conserved in plakin family members, important for mechano-sensing [41] and have been suggested to not bind the canonical PXXP-binding groove [42], correlating with our suggested binding motif in hnRNP G (PPP) (Supplementary Fig. 4). *COL2A* is important for collagen production, bones, tissue, and sensory development [43]. *ZNF805* is a transcription factor involved in RNA polymerase II transcription, consistent with the overrepresentation of transcription factors seen in our top 100 DEGs. *LYSMD3* encodes a receptor on human airway epithelial cells, important for immune response to airway pathogens [44]. *TTN* is an important muscle protein in the heart, associated to a number of different disorders including neuromuscular disorders [45] and respiratory failure [46]. *PCDHA10* encodes protocadherin α expressed in brain and eye [47], important in neurodevelopment [48].

*RBMX* has previously been associated with Shashi syndrome (OMIM #300238) [11] caused by a p.Glu346fs variant [11], leading to disruption of the RGG/RG motif and thus aberrant p53 activation and neuronal differentiation [10]. Patients with Gustavson syndrome have minor shared phenotypes with Shashi syndrome, severe phenotypes such as structural brain abnormalities, epilepsy, severe vision defects, hearing loss, and early death are not present in Shashi patients. This suggests that different variants disrupting different hnRNP G domains lead to distinct phenotypes. Furthermore, Gustavson syndrome has a larger phenotypic overlap with other hnRNP-associated neurodevelopmental disorders, including the most commonly reported symptoms such as intellectual disability, seizures, hypotonia and severe structural brain abnormalities [2] (Supplementary Table 4). To our knowledge, death has only been described in one patient with a hnRNP-associated neurodevelopmental disorder before (15 years old female), associated with a heterozygous predicted splice variant in hnRNPK (NM_031263.2;c.258-3 C > T) [2]. However, other likely gene disrupting variants in hnRNPK were not lethal, and missense variants in the same gene and/or domain result in the same severity as likely gene disrupting variants, indicating no clear genotype-phenotype correlation in this gene. Thus, Gustavson syndrome is the most severe intellectual disability syndrome described in this gene family so far. Early death is consistent with knock-down experiments performed in zebrafish [7] and depletion of pLoF variants in *RBMX* in the general human population (Fig. 1C), which may indicate that the p.(Pro162del) causes loss of hnRNP G function.

Human retroposon-derived RBMX Like 1 (*RBMXL1*) and RBMX Like 9 (*RBMXL9*) have 96% sequence similarity to *RBMX* and are expressed in various tissues including brain [49]. The gene retrocopies may potentially replace or compensate hnRNP G function; however, the tri-proline region of interest (aa 160–162; P38159) are disrupted in the retrocopies and therefore unlikely to compensate for the *RBMX* variant effect in our patients (Supplementary Fig. 5). Moreover, we did not identify any disease-modifying or additional potential disease-causing variants in *RBMXL1* and *RBMXL9* as likely contributions to Gustavson syndrome. However, more studies are needed to confirm the function of the retrocopies and we would like to highlight the possibility that phenotypic differences to Shashi syndrome may be due to variant rescue by gene retrocopy/ies.

In conclusion, an in-frame deletion in *RBMX* (c.484_486del, p.(Pro162del)) leads to Gustavson syndrome. Protein interaction analyses revealed the first indication that amino acid 156–169 of hnRNP G possibly is an SH3-binding motif and that the variant could reduce binding affinity to SH3-domains. Transcriptomics analyses indicated that the variant region has important functions in transcription by RNA polymerase II.

### Supplementary information

Supplementary material 1

Supplementary material 2

Supplementary material 3

Supplementary methods 1

Supplementary methods 2

Supplementary methods 3

Supplementary data 1

Supplementary figure 1

Supplementary figure 2

Supplementary figure 3

Supplementary figure 4

Supplementary figure 5

Supplementary table 1

Supplementary table 2A

Supplementary table 2B

Supplementary table 2C

Supplementary table 3A

Supplementary table 3B

Supplementary table 3C

Supplementary table 4A

Supplementary table 4B

## Data Availability

The data generated and analyzed during the current study are not publicly available due to Swedish legislation. Data from transcriptomics analysis is available upon request.
