# Supplementary material for: Gustavson syndrome is caused by an in-frame deletion in RBMX associated with potentially disturbed SH3 domain interactions
Source: Eur J Hum Genet. 2023 Jun 5;32(3):333–41. doi: 10.1038/s41431-023-01392-y (PMC10923852; doi:10.1038/s41431-023-01392-y)
Supplement: Supplementary file 1 — Supplementary material 1 [file 41431_2023_1392_MOESM1_ESM.docx]

**Supplementary material 1.** PCR protocols and primers used in the experiments. NA = not applicable.

| **Experiment** | **Kit or protocol** | **Primer name** | **Forward** | **Reverse** | **Thermocycler conditions** |
| --- | --- | --- | --- | --- | --- |
| Segregation analysis | 20µl reaction: 250nM dNTPs, 1µM of each primer, 1X PCR reaction buffer and 1U Taq polymerase (AmpliTaq Gold DNA polymerase with buffer I, Thermo Fisher Scientific, Baltics UAB, Vilnius, Lithuania) | RBMX_DNA | GATGCAGATGACGGTGGAT | CCTCTCAATTCTTTGTGTTACGG | 95°C 5 min, 20x (95°C 20 sec, 65-55°C 30 sec, 72°C 60 sec) and 25x (95°C 20 sec, 55°C 30 sec, 72°C 60 sec) 4°C hold |
| X-chromosome inactivation analysis (RNA) | 20µl reaction: 250nM dNTPs, 1µM of each primer, 1X PCR reaction buffer and 1U Taq polymerase (AmpliTaq Gold DNA polymerase with buffer I, Thermo Fisher Scientific, Baltics UAB, Vilnius, Lithuania) | RBMX_RNA | AAACATGGTTGAAGCAGATCG | CCATCATCTCTTGGGGACAA | 95°C 5 min, 20x (95°C 20 sec, 65-55°C 30 sec, 72°C 60 sec) and 25x (95°C 20 sec, 55°C 30 sec, 72°C 60 sec) 4°C hold |
| X -hromosome inactivation analysis (*AR*, DNA) | LongRange PCR kit (Qiagen, Hilden, Germany) according to manufacturer’s instructions | AR_XCI | Primer as described before (Allen et al., 1992) | Primer as described before (Allen et al., 1992) | Thermal cycler conditions as described before (Allen et al., 1992) |
| X-chromosome inactivation analysis (*RP2*, DNA) | 20µl reaction: 250nM dNTPs, 1µM of each primer, 1X PCR reaction buffer and 1U Taq polymerase. | RP2_XCI | Primer as described before (Machado et al., 2014) | Primer as described before (Machado et al., 2014) | Thermal cycler conditions as described before (Machado et al., 2014) |
| *RBMX* splicing investigation | 20µl reaction: 250nM dNTPs, 1µM of each primer, 1X PCR reaction buffer and 1U Taq polymerase (AmpliTaq Gold DNA polymerase with buffer I, Thermo Fisher Scientific, Baltics UAB, Vilnius, Lithuania) | RBMX_Splicing_1 | AAACATGGTTGAAGCAGATCG | CTTCTGCCTCCCCCTCTATC | 95°C 5 min, 20x (95°C 20 sec, 65-55°C 30 sec, 72°C 90 sec) and 25x (95°C 20 sec, 55°C 30 sec, 72°C 90 sec) 4°C hold |
| *RBMX* splicing investigation | 20µl reaction: 250nM dNTPs, 1µM of each primer, 1X PCR reaction buffer and 1U Taq polymerase (AmpliTaq Gold DNA polymerase with buffer I, Thermo Fisher Scientific, Baltics UAB, Vilnius, Lithuania) | RBMX_Splicing_2 | TGGAAAGTCATTAGATGGAAAAGC | TTGGTCCAAAGTTTTGTTTGTTT | 95°C 5 min, 20x (95°C 20 sec, 65-55°C 30 sec, 72°C 90 sec) and 25x (95°C 20 sec, 55°C 30 sec, 72°C 90 sec) 4°C hold |
| Mini-gene construct | LongRange PCR kit (Qiagen, Hilden, Germany) according to manufacturer’s instructions | Outer_Cloning | TGGCTTTTGGTTTTGGCTAC | CAACTGGGTGGGAGGTTTTA | 93°C 3 min, 35x (93°C 15 sec, 55°C 30 sec, 68°C 4 min), 4°C hold |
| Mini-gene construct | LongRange PCR kit (Qiagen, Hilden, Germany) according to manufacturer’s instructions | Inner_Cloning | GCATGAATTCGTCATTAGATGGAAAAGCCATCAA | GCATGGATCCGCTGTCTTTAGTAGAATACCCATCA | 93°C 3 min, 35x (93°C 15 sec, 55°C 30 sec, 68°C 3 min), 4°C hold |
| Mini-gene splicing assay | LongRange PCR kit (Qiagen, Hilden, Germany) according to manufacturer’s instructions | RBMX_RNA_Cells | AGGTGGAACAAGCCACCAAA | CATCTCTACGAGAGGGCAGC | 93°C 3 min, 35x (93°C 15 sec, 58°C 30 sec, 68°C 35 sec), 4°C hold |
