# Supplementary material for: Gustavson syndrome is caused by an in-frame deletion in RBMX associated with potentially disturbed SH3 domain interactions
Source: Eur J Hum Genet. 2023 Jun 5;32(3):333–41. doi: 10.1038/s41431-023-01392-y (PMC10923852; doi:10.1038/s41431-023-01392-y)
Supplement: Supplementary file 2 — Supplementary material 2 [file 41431_2023_1392_MOESM2_ESM.docx]

**Supplementary material 2.** HnRNP G peptides (P38159) used in the fluorescence polarization assay.

| **Name** | **Amino acid sequence** |
| --- | --- |
| hnRNP G aa 156-169, wildtype | 156-RSGGPPPKRSAPSGy-169 |
| hnRNP G aa 156-169, △P162 | 156-RSGGPPKRSAPSGy-169 |
| hnRNP G aa 156-169, wildtype FITC | 156-RSGGPPPKRSAPSGy-169 |
| hnRNP G aa 156-169, △P162 FITC | 156-RSGGPPKRSAPSGy-169 |
| hnRNP G aa 150-170, wildtype | 150-KRGPPPRSGGPPPKRSAPSGPy-170 |
| hnRNP G aa 150-170, △P162 | 150-KRGPPRSGGPPPKRSAPSGPy-170 |
| hnRNP G aa 150-170, wildtype FITC | 150-KRGPPPRSGGPPPKRSAPSGPy-170 |
| hnRNP G aa 150-170, △P162 FITC | 150-KRGPPRSGGPPPKRSAPSGPy-170 |
