# Supplementary material for: Gustavson syndrome is caused by an in-frame deletion in RBMX associated with potentially disturbed SH3 domain interactions
Source: Eur J Hum Genet. 2023 Jun 5;32(3):333–41. doi: 10.1038/s41431-023-01392-y (PMC10923852; doi:10.1038/s41431-023-01392-y)
Supplement: Supplementary file 3 — Supplementary material 3 [file 41431_2023_1392_MOESM3_ESM.docx]

**Supplementary material 3.** The constructs used in transcriptomics experiment, obtained from GenScript (Leiden, Netherlands).

>WT_pcDNA3.1(+)-C-eGFP, *RBMX* insert

ATGGTTGAAGCAGATCGCCCAGGAAAGCTCTTCATTGGTGGGCTTAATACGGAAACAAATGAGAAAGCTCTTGAAGCAGTATTTGGCAAATATGGACGAATAGTGGAAGTACTCTTGATGAAAGACCGTGAAACCAACAAATCAAGAGGATTTGCTTTTGTCACCTTTGAAAGCCCAGCAGACGCTAAGGATGCAGCCAGAGACATGAATGGAAAGTCATTAGATGGAAAAGCCATCAAGGTGGAACAAGCCACCAAACCATCATTTGAAAGTGGTAGACGTGGACCGCCTCCACCTCCAAGAAGTAGAGGCCCTCCAAGAGGTCTTAGAGGTGGAAGAGGAGGAAGTGGAGGAACCAGGGGACCTCCCTCACGGGGAGGACACATGGATGACGGTGGATATTCCATGAATTTTAACATGAGTTCTTCCAGGGGACCACTCCCAGTAAAAAGAGGACCACCACCAAGAAGTGGGGGTCCTCCTCCTAAGAGATCTGCACCTTCAGGACCAGTTCGCAGTAGCAGTGGAATGGGAGGAAGAGCTCCTGTATCACGTGGAAGAGATAGTTATGGAGGTCCACCTCGAAGGGAACCGCTGCCCTCTCGTAGAGATGTTTATTTGTCCCCAAGAGATGATGGGTATTCTACTAAAGACAGCTATTCAAGCAGAGATTACCCAAGTTCTCGTGATACTAGAGATTATGCACCACCACCACGAGATTATACTTACCGTGATTATGGTCATTCCAGTTCACGTGATGACTATCCATCAAGAGGATATAGCGATAGAGATGGATATGGTCGTGATCGTGACTATTCAGATCATCCAAGTGGAGGTTCCTACAGAGATTCATATGAGAGTTATGGTAACTCACGTAGTGCTCCACCTACACGAGGGCCCCCGCCATCTTATGGTGGAAGCAGTCGCTATGATGATTACAGCAGCTCACGTGACGGATATGGTGGAAGTCGAGACAGTTACTCAAGCAGCCGAAGTGATCTCTACTCAAGTGGTCGTGATCGGGTTGGCAGACAAGAAAGAGGGCTTCCCCCTTCTATGGAAAGGGGGTACCCTCCTCCACGTGATTCCTACAGCAGTTCAAGCCGCGGAGCACCAAGAGGTGGTGGCCGTGGAGGAAGCCGATCTGATAGAGGGGGAGGCAGAAGCAGATACTAG

>Mut_pcDNA3.1(+)-C-eGFP, *RBMX* insert

ATGGTTGAAGCAGATCGCCCAGGAAAGCTCTTCATTGGTGGGCTTAATACGGAAACAAATGAGAAAGCTCTTGAAGCAGTATTTGGCAAATATGGACGAATAGTGGAAGTACTCTTGATGAAAGACCGTGAAACCAACAAATCAAGAGGATTTGCTTTTGTCACCTTTGAAAGCCCAGCAGACGCTAAGGATGCAGCCAGAGACATGAATGGAAAGTCATTAGATGGAAAAGCCATCAAGGTGGAACAAGCCACCAAACCATCATTTGAAAGTGGTAGACGTGGACCGCCTCCACCTCCAAGAAGTAGAGGCCCTCCAAGAGGTCTTAGAGGTGGAAGAGGAGGAAGTGGAGGAACCAGGGGACCTCCCTCACGGGGAGGACACATGGATGACGGTGGATATTCCATGAATTTTAACATGAGTTCTTCCAGGGGACCACTCCCAGTAAAAAGAGGACCACCACCAAGAAGTGGGGGTCCTCCTAAGAGATCTGCACCTTCAGGACCAGTTCGCAGTAGCAGTGGAATGGGAGGAAGAGCTCCTGTATCACGTGGAAGAGATAGTTATGGAGGTCCACCTCGAAGGGAACCGCTGCCCTCTCGTAGAGATGTTTATTTGTCCCCAAGAGATGATGGGTATTCTACTAAAGACAGCTATTCAAGCAGAGATTACCCAAGTTCTCGTGATACTAGAGATTATGCACCACCACCACGAGATTATACTTACCGTGATTATGGTCATTCCAGTTCACGTGATGACTATCCATCAAGAGGATATAGCGATAGAGATGGATATGGTCGTGATCGTGACTATTCAGATCATCCAAGTGGAGGTTCCTACAGAGATTCATATGAGAGTTATGGTAACTCACGTAGTGCTCCACCTACACGAGGGCCCCCGCCATCTTATGGTGGAAGCAGTCGCTATGATGATTACAGCAGCTCACGTGACGGATATGGTGGAAGTCGAGACAGTTACTCAAGCAGCCGAAGTGATCTCTACTCAAGTGGTCGTGATCGGGTTGGCAGACAAGAAAGAGGGCTTCCCCCTTCTATGGAAAGGGGGTACCCTCCTCCACGTGATTCCTACAGCAGTTCAAGCCGCGGAGCACCAAGAGGTGGTGGCCGTGGAGGAAGCCGATCTGATAGAGGGGGAGGCAGAAGCAGATACTAG
