# Supplementary material for: Gustavson syndrome is caused by an in-frame deletion in RBMX associated with potentially disturbed SH3 domain interactions
Source: Eur J Hum Genet. 2023 Jun 5;32(3):333–41. doi: 10.1038/s41431-023-01392-y (PMC10923852; doi:10.1038/s41431-023-01392-y)
Supplement: Supplementary file 4 — Supplementary methods 1 [file 41431_2023_1392_MOESM4_ESM.docx]

**Supplementary methods 1. Mini-gene splicing assay; mini-gene construct, purification, cell transfection, RNA extraction and cDNA synthesis.**

The mini-gene construct was created by performing a nested PCR with PCR protocols described in **Supplementary material 1**. The first PCR was performed using 10ng DNA from a heterozygous individual (III:30) and the Outer_Cloning primers, and the second PCR was performed with 1µl PCR product as input and the Inner_Cloning primers (containing restriction sites for EcoRI and BamHI). Both the insert and pEGFP-C1 were digested with EcoRI and BamHI (Fast digest enzymes, Thermo Fisher Scientific Baltics UAB, Vilnius, Lithuania), gel purified using Nucleospin Gel and PCR clean-up kit (Machery Nagel, Düren, Germany) and ligated for 2h, 22°C using Rapid DNA Ligation Kit (Thermo Fisher Scientific Baltics UAB, Vilnius, Lithuania). Transformation was performed using One Shot TOP10 Chemically Competent *E. coli* (Invitrogen, Carlsbad, CA, USA). One vial of *E. coli* was split into two reactions and 10ng DNA (1:3 resp. 1:5 ligation, vector:insert) was added per tube and gently mixed. The bacteria were incubated on ice for 30 min, heat shocked at 42°C for 45 sec and placed on ice for 2 min. Two ml room temperature LB medium was added and the reactions were then placed in a shaking incubator (200 rpm) for 1 hour at 37°C. DNA was extracted from colonies using GeneJET Plasmid Miniprep Kit (Thermo Fisher Scientific Baltics UAB, Vilnius, Lithuania) according to protocol and the genotype was verified by Sanger sequencing using the Mix2seq kit (Eurofin Genomics, Ebersberg, Germany).

HeLa (passage 32) and SH-SY5Y (passage 54) cells were cultured separately in Dulbecco's Modified Eagle Medium with 10% fetal bovine serum and 1% antibiotic myotic at 37°C and 5% CO2. A total of 2.3 x 106 of each cell line were seeded in 6-well plates and grown over-night. The transfection was performed in triplicates using 3µg of plasmid DNA per well with the variant and wildtype respectively using jetPEI® DNA transfection reagent according to manufacturer’s instructions (Polyplus-transfection, San Diego, CA, USA). The cells were incubated for 48h and RNA was isolated using Trizol Reagent (Invitrogen, Carlsbad, CA, USA) and purified using RNeasy Micro Kit (Qiagen, Hilden, Germany) according to manufacturer’s instructions. cDNA synthesis was performed as described before. PCR was performed using 50ng of cDNA as input, the RBMX_RNA_Cells primers and PCR protocol described in **Supplementary material 1**. Cycle sequencing was performed using as previously described.
