# Supplementary material for: Gustavson syndrome is caused by an in-frame deletion in RBMX associated with potentially disturbed SH3 domain interactions
Source: Eur J Hum Genet. 2023 Jun 5;32(3):333–41. doi: 10.1038/s41431-023-01392-y (PMC10923852; doi:10.1038/s41431-023-01392-y)
Supplement: Supplementary file 5 — Supplementary methods 2 [file 41431_2023_1392_MOESM5_ESM.docx]

**Supplementary methods 2. Purification of SH3 protein domains.**

SH3 domains were expressed by transforming expression constructs into *E. coli* (BL21-DE3 GOLD) and grown in LB media with 50 μg/ml ampicillin (ASAP1) or 50 μg/ml kanamycin (BIN1) at 37°C until reaching an OD_600_ of 0.6. Protein expression was induced with 1mM isopropyl-β-thiogalactopyranoside and the protein expression proceeded overnight at 18 °C. Cells were harvested and resuspended in lysis buffer (50 mM Tris/HCl pH 7.8, 10 µg/mL DNase I and RNase A (Hoffman-La Roche, Switzerland), 300 mM NaCl, 4 mM MgCl_2_, 2 mM CaCl_2_ and cOmplete EDTA-free Protease Inhibitor Cocktail (Hoffman-La Roche)) and lysed by sonication. Cell lysate was clarified by centrifugation at 20,000 g for 40 min, filtered and transferred to Glutathione Sepharose 4 Fast Flow media (GE Healthcare). After 30-minute incubation, glutathione media was washed with wash buffer (50 mM Tris/HCl pH 7.8 300 mM NaCl, 4 mM DTT), eluted with elution buffer (50 mM Tris/HCl pH 7.8 300 mM NaCl, 4 mM DTT, 10 mM reduced glutathione), and the GST-tag was cleaved overnight with PreScission protease (BIN1) or Thrombin (ASAP1). Next day the cleaved protein was applied to a nickel Sepharose column to remove the tag, and pure protein was dialyzed into 50 mM potassium phosphate buffer pH 7.4, and concentrated using the Amicon ultra 15ml centrifugal filters (3kDa cutoff, Merck Millipore, UFC900308). Purity of the sample was analyzed by SDS-PAGE and its identity confirmed by MALDI-TOF mass spectrometry. The sample was then frozen until fluorescence polarization experiments were performed.
