# Supplementary material for: Gustavson syndrome is caused by an in-frame deletion in RBMX associated with potentially disturbed SH3 domain interactions
Source: Eur J Hum Genet. 2023 Jun 5;32(3):333–41. doi: 10.1038/s41431-023-01392-y (PMC10923852; doi:10.1038/s41431-023-01392-y)
Supplement: Supplementary file 6 — Supplementary methods 3 [file 41431_2023_1392_MOESM6_ESM.docx]

**Supplementary methods 3. Localization and expression of hnRNP G protein. Permutation test for mean expression of mutant and wildtype cell populations.**

A µ-Slide 8 Well (ibidi, Gräfelfing, Germany) was coated with Poly-L-ornithine hydrobromide (15mg/ml solution; Sigma-Aldrich, Spruce Street, Saint Louis, MO, USA) for 30 min at RT and then Laminin from Engelbreth-Holm-Swarm murine sarcoma basement membrane (1:500 dilution in PBS; Sigma-Aldrich, Spruce Street, Saint Louis, MO, USA) over night. Approximately 60 000 SH-SY5Y cells (passage 69) were seeded and transient transfection was performed using pcDNA3.1(+)-C-eGFP plasmid constructs containing the *RBMX* cDNA sequence with and without the variant (c.484_486del, p.(Pro162del)) (**Supplementary material 3**) and transfected according to manufacturer’s instructions (Polyplus-transfection, San Diego, CA, USA) for 48h. After 72h the cells were treated with VECTASHIELD® Antifade Mounting Medium with DAPI (Vector Laboratories, Mowry Avenue, Newark CA, USA). Imaging was performed using Zeiss LSM700 Confocal Microscope, at 40x magnification. Protein expression was measured from images taken with EVOS FL Auto 2 (Fisher Scientific, Willow Creek, Eugene, OR, USA) using ImageJ (Rasband, W.S., ImageJ, U. S. National Institutes of Health, Bethesda, Maryland, USA, https://imagej.nih.gov/ij/, 1997-2018). The total intensity was measured for a total of 68 cells expressing the Pro162del construct and 91 cells expressing the wild-type construct. Rstudio (v2022.12.0) was used to perform permutation test (N=10,000) and to plot the histogram.
