# Supplementary material for: Gustavson syndrome is caused by an in-frame deletion in RBMX associated with potentially disturbed SH3 domain interactions
Source: Eur J Hum Genet. 2023 Jun 5;32(3):333–41. doi: 10.1038/s41431-023-01392-y (PMC10923852; doi:10.1038/s41431-023-01392-y)
Supplement: Supplementary file 7 — Supplementary data 1 [file 41431_2023_1392_MOESM7_ESM.docx]

**Supplementary data 1. Patient descriptions of the affected individuals IV:23, V:3 and V:7.**

V:7 was born after a spontaneous delivery at gestational week 37 with a birth weight of 3125g. Reduced fetal movements was noted during pregnancy and microcephaly was diagnosed in the third trimester. His healthy non-consanguineous parents have an older unaffected daughter. The mother has three children in an earlier relationship, two healthy daughters and a son born with microcephaly, small chin, short stature, small eyes, puffy eyelids, high palate and hypotonia who died at the age of 4 months (V:3). In addition, V:7s maternal uncle (IV:23), born 1972, had microcephaly, short stature and died at age 1.5 months. Clinical findings of V:7 at birth included microcephaly, hypotonia, bilateral pes equinovarus and claw-like positioned hands. He was diagnosed with autism spectrum disorder, ventricular septum defect, and required surgical treatment due to a left sided inguinal herniation. Magnetic resonance imaging showed lissencephaly and pachygyria. Dysmorphic features include bitemporal narrowing, wide mouth, puffy eyelids, broad mouth, high palate, two hemangiomas, microcephaly, short stature and overweight. He is non ambulant and has profound intellectual disability, seizures, impaired hearing, limited movements and cannot communicate (**Figure 2**).
