# Supplementary figures and images for: Gustavson syndrome is caused by an in-frame deletion in RBMX associated with potentially disturbed SH3 domain interactions
Source: Eur J Hum Genet. 2023 Jun 5;32(3):333–41. doi: 10.1038/s41431-023-01392-y (PMC10923852; doi:10.1038/s41431-023-01392-y)

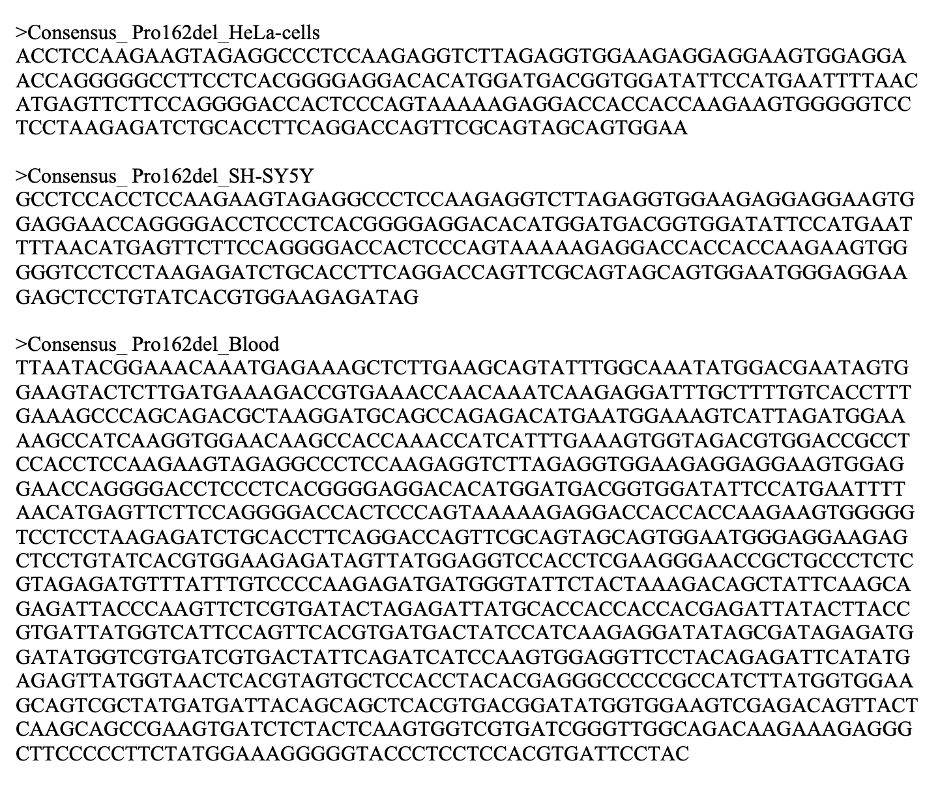

Supplement: Supplementary file 9 — Supplementary figure 2 [file 41431_2023_1392_MOESM9_ESM.png]

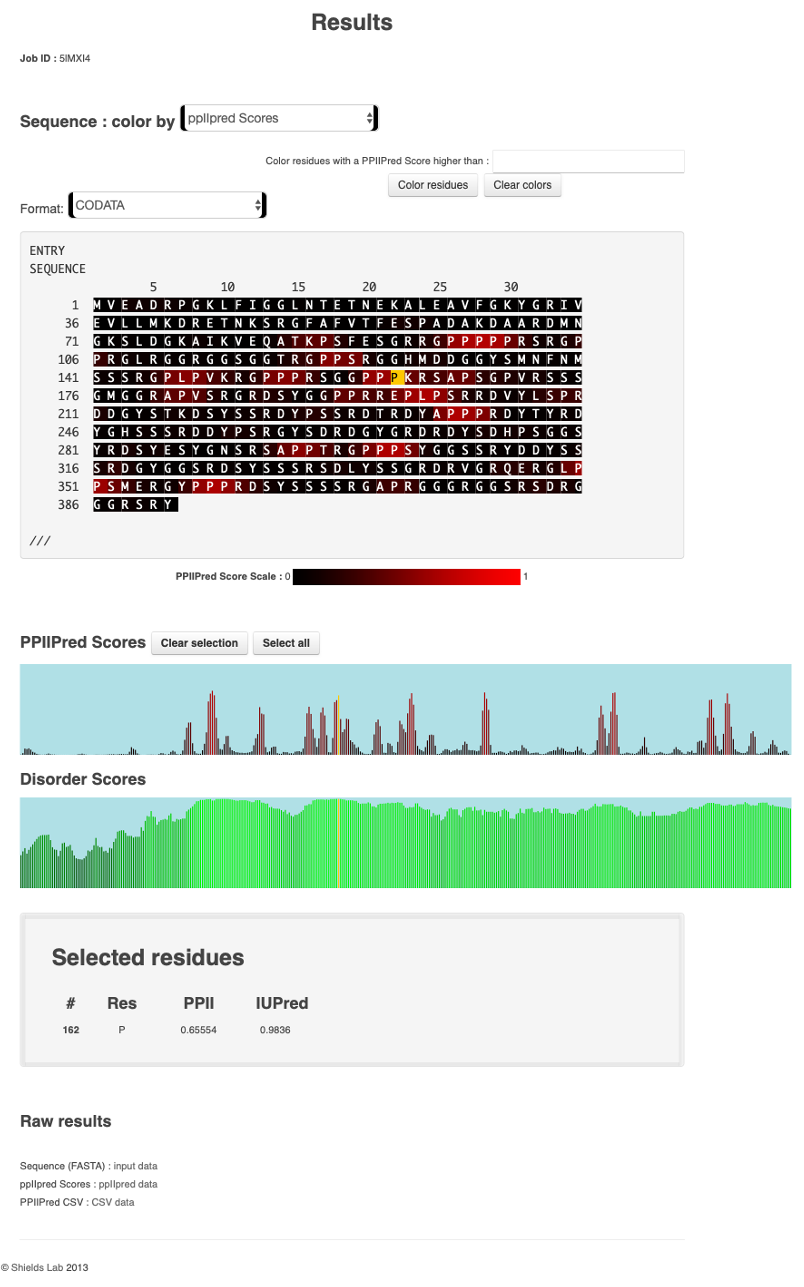

Supplement: Supplementary file 11 — Supplementary figure 4 [file 41431_2023_1392_MOESM11_ESM.png]

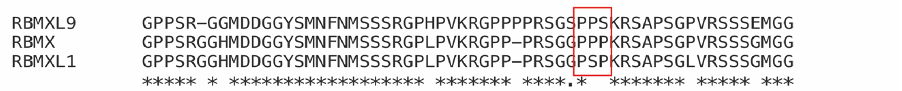

Supplement: Supplementary file 12 — Supplementary figure 5 [file 41431_2023_1392_MOESM12_ESM.png]
