# Supplementary material for: Gustavson syndrome is caused by an in-frame deletion in RBMX associated with potentially disturbed SH3 domain interactions
Source: Eur J Hum Genet. 2023 Jun 5;32(3):333–41. doi: 10.1038/s41431-023-01392-y (PMC10923852; doi:10.1038/s41431-023-01392-y)
Supplement: Supplementary file 13 — Supplementary table 1 [file 41431_2023_1392_MOESM13_ESM.pdf]

Supplementary table 1. Sanger sequencing confirmed segregation of the *RBMX* variant (NM_002139.4; c.484_486del, p.(Pro162del)) in 36 samples of the Gustavson family. Wt = wildtype, Het = heterozygote, Hemi = hemizygous.

**ID**	***RBMX* genotype (p.(Pro162del))**
II:1	Wt
II:3	Het
II:5	Het
II:7	Wt
II:8	Het
II:10	Wt
II:12	Wt
III:2	Wt
III:4	Het
III:6	Het
III:7	Wt
III:9	Wt
III:11	Wt
III:14	Het
III:17	Wt
III:18	Het
III:19	Wt
III:20	Het
III:22	Het
III:28	Wt
III:29	Wt
III:33	Wt
IV:1	Wt
IV:2	Het
IV:5	Hemi
IV:7	Wt
IV:8	Wt
IV:9	Wt
IV:15	Wt
IV:19	Hemi
IV:21	Wt
IV:23	Hemi
IV:26	Het
V:2	Wt
V:7	Hemi
